# Mitochondrial protein import in trypanosomatids: Variations on a theme or fundamentally different?

**DOI:** 10.1371/journal.ppat.1007351

**Published:** 2018-11-29

**Authors:** André Schneider

**Affiliations:** Department of Chemistry and Biochemistry, University of Bern, Freiestrasse, Bern, Switzerland; University at Buffalo School of Medicine and Biomedical Sciences, UNITED STATES

Mitochondria perform many important functions. Their origin can be traced back to an endosymbotic event between an archaeal host cell and an α-proteobacteria approximately 2 billion years ago [1]. Subsequently, the endosymbiont was converted into an organelle, which learned to import cytosolic proteins, a feat present-day endosymbiontic bacteria are not capable of. Today, the large majority of mitochondrial proteins are encoded in the nucleus, synthesized in the cytosol, and finally imported across the outer and/or the inner mitochondrial membranes. Protein import was one of the first—if not the first—mitochondria-specific trait to evolve. Because mitochondria are monophyletic, the expectation was that the machineries that mediate mitochondrial protein import would be largely conserved. Work in trypanosomes and other organisms in recent years has shown that this is not the case [2, 3]. It is the aim of this review to summarize where we find major deviations in the trypanosomal mitochondrial protein import machineries when compared to the best-studied system, that of the yeast *Saccharomyces cerevisiae*.

## How different are the mitochondrial protein import receptors?

**Fig 1:**
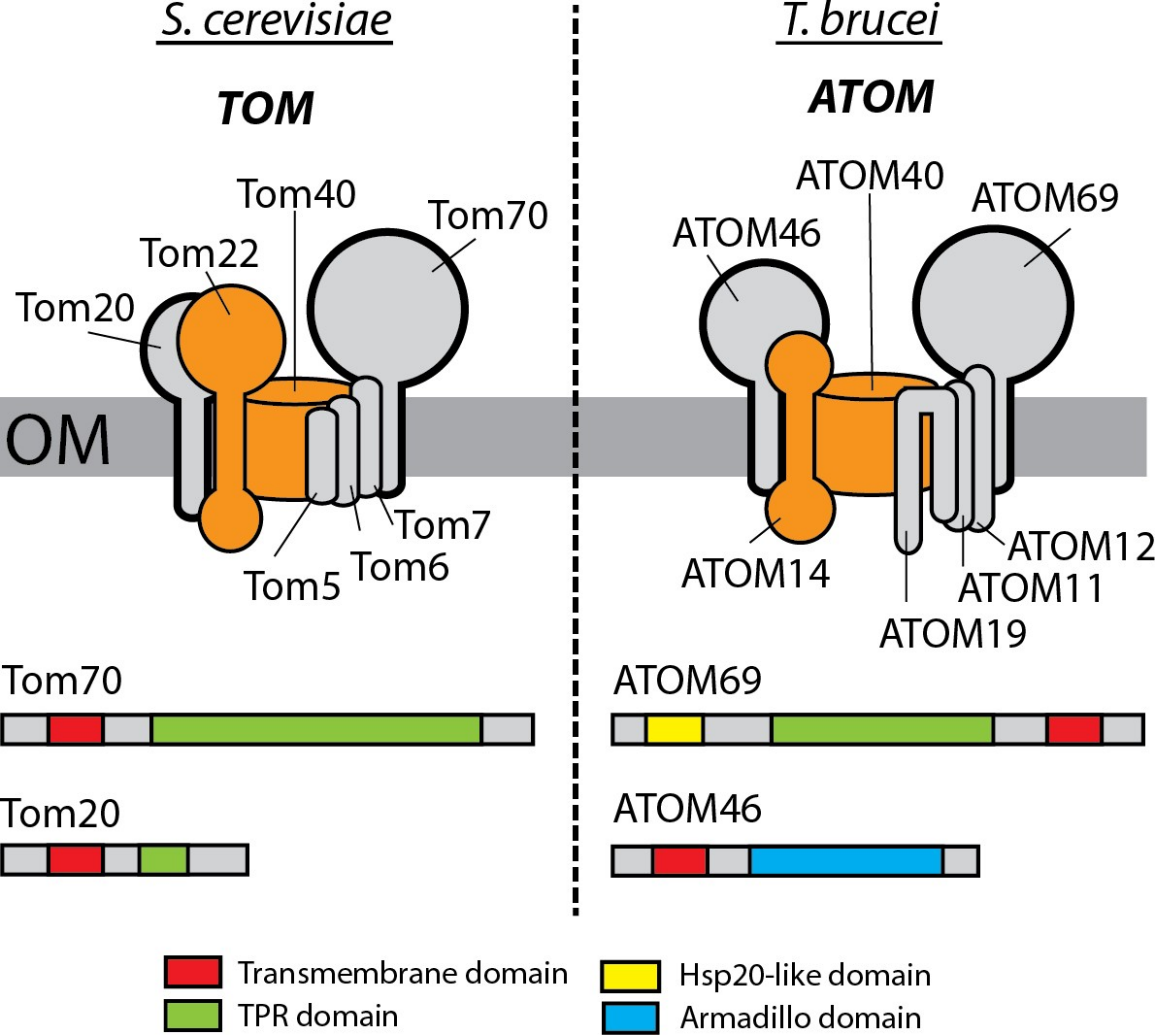
Comparison of the main OM protein translocases between *S*. *cerevisiae* (TOM) and *Trypanosoma brucei* (ATOM). Tom40/ATOM40 and Tom22/ATOM14 are orthologues and are indicated in orange. Other subunits are shown in light gray. The protein import receptor pairs in yeast (Tom20/Tom70) and trypanosomes (ATOM46/ATOM69) are indicated in bold. They are not evolutionarily related but share the same function. The domain structures of the yeast and the trypanosomal receptor pairs are indicated at the bottom. The depiction of the ATOM complex is schematic; its exact architecture remains to be elucidated. ATOM, atypical translocase of the outer membrane; Hsp20, heat shock protein 20; OM, outer membrane; Tom, translocase of the outer membrance; TPR, tetratricopeptide repeat.

There is ample evidence that the mitochondrial targeting signals between trypanosomal and yeast proteins are functionally exchangeable [3]. It came, therefore, as a surprise that the outer membrane (OM) receptors recognizing these signals were not conserved (Fig 1). Both systems have two primary protein import receptors each, termed Tom20 and Tom70 in yeast [4, 5] and ATOM46 and ATOM69 in trypanosomes [3, 6]. In both organisms, they are associated with the main protein translocase of the mitochondrial OM, termed TOM complex in yeast and ATOM complex in trypanosomes (Fig 1). The acronym ATOM was originally defined as "archaic TOM" [7]. However more recent work showed that its core component ATOM40 is in fact a hard to recognize orthologue of Tom40 [8] which is why would like to rename ATOM to "atypical TOM".

Each receptor type within the same organism has distinct but in part overlapping substrate specificities: Tom20 seems to recognize mainly presequence-containing proteins, whereas Tom70 is specialized for hydrophobic proteins having internal targeting sequences. Moreover, Tom70 also has a chaperone-like function in preventing precursor proteins from premature folding prior to import [9].

Functionally, yeast Tom20 resembles trypanosomal ATOM46, and Tom70 behaves like ATOM69, although the precise substrate specificities of the two trypanosomal receptors have not yet been determined. Despite their functional similarities, Tom20 and ATOM46 do not share sequence similarity, and the same is the case for the Tom70/ATOM69 pair. Tom20 and ATOM46 are anchored in the OM by a single N-terminal transmembrane domain but have very different molecular weights. Moreover, whereas Tom20 contains a single tetratricopeptide repeat (TPR) motif, ATOM46 contains armadillo repeats (Fig 1). Superficially, Tom70 is similar to ATOM69 because both contain numerous TPR motifs and also share the same molecular weight. However, in contrast to Tom70, which is N-terminally anchored in the OM, the transmembrane domain of ATOM69 is at the C-terminus, resulting in an inverted topology of the two proteins [3, 6]. Furthermore, ATOM69 contains an Hsp20 domain that is absent from Tom70 (Fig 1). Therefore, yeast Tom20/Tom70 and trypanosomal ATOM46/ATOM69 are functional analogues that arose independently by convergent evolution [3, 6].

Tom20 and Tom70 orthologues are found in opisthokonts, the eukaryotic supergroup that includes fungi and metazoans, and—at least in the case of Tom70—are found also in its sister group the amoebozoans [10]. ATOM46 and ATOM69 were so far only found in kinetoplastids, and further analysis is required to determine whether they also occur in other excavates.

Mitochondria of plants, which belong to the supergroup of the Archeaplastidae, have a receptor pair Tom20/OM64 that is distinct from both yeast Tom20/Tom70 as well as from ATOM46/ATOM69 [11, 12]. Therefore, even though yeast and plant Tom20 bear the same name, they are not orthologues because the plant protein in contrast to the yeast one is C-terminally anchored in the OM.

In summary, these findings suggest that the mitochondrial protein import receptors evolved only after the eukaryotes started to diverge into different clades [3, 6, 11, 13].

## A fundamentally different OM protein biogenesis factor in *T*. *brucei*?

**Fig 2:**
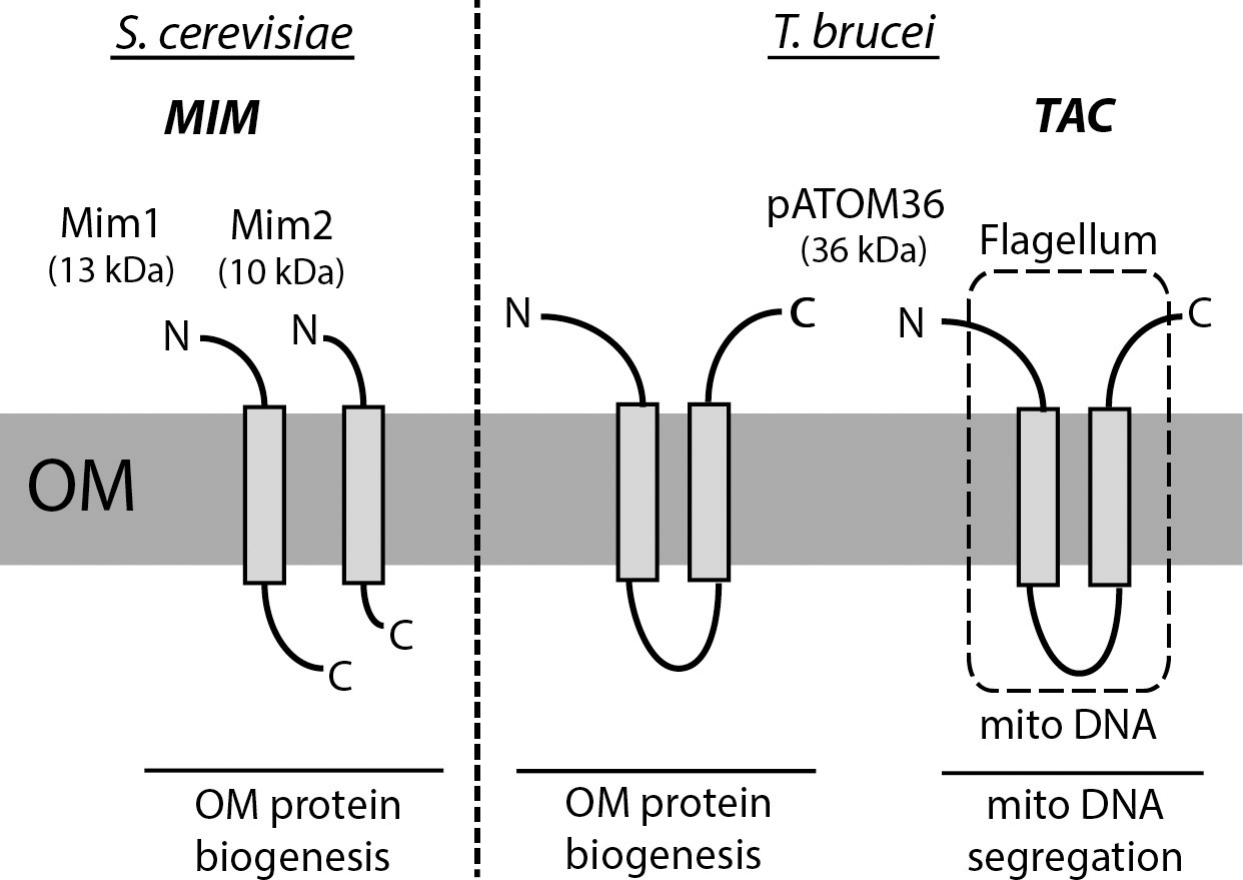
Comparison between the MIM complex of *S*. *cerevisiae* and pATOM36 of *T*. *brucei*. The two complexes that are required for the biogenesis of a subset of OM proteins including subunits of the TOM and ATOM complexes, respectively. The MIM complex consists of Mim1 and Mim2, neither of which shows sequence similarity with pATOM36. Although pATOM36 is an integral membrane protein, its two predicted transmembrane domains have not yet been confirmed experimentally. pATOM36 is dually localized all over the OM and in the TAC (box with dashed lines) that links the mitochondrial DNA with the basal body of the flagellum. Therefore, pATOM36 has an essential function in OM protein biogenesis and as an essential component of the TAC in the segregation of the replicated single unit kDNA of *T*. *brucei*. kDNA, kinetoplast DNA; MIM, mitochondrial import machinery; OM, outer membrane; pATOM36, peripheral ATOM of 36 kDa; TAC, tripartite attachment complex.

Biogenesis of N-terminally anchored OM proteins of yeast, some of which are subunits of the TOM complex, requires the mitochondrial import machinery (MIM) complex consisting of Mim1 and Mim2, respectively [14–16]. The MIM complex is specific to fungi and facilitates the insertion and/or assembly of N-terminally anchored OM proteins by an as yet unknown mechanism. Recently, a protein, termed pATOM36, has been characterized that carries out the same function in trypanosomes [17] (Fig 2). Its depletion reduced the levels of a selected set of mitochondrial OM proteins. pATOM36 was shown to mediate assembly of the ATOM complex by facilitating its association with the protein import receptor ATOM46 [18]. Moreover, for POMP10, an OM protein of unknown function, pATOM36 was required for membrane insertion [19]. pATOM36 is also present in the tripartite attachment complex (TAC) that connects the single-unit mitochondrial genome of trypanosomes to the basal body of the flagella [18] (Fig 2). Therefore, in contrast to the MIM complex, pATOM36 has a dual function in the biogenesis of OM proteins as well as in the segregation of the replicated mitochondrial genome.

The fungi-specific Mim1 (13 kD) and Mim2 (10 kD) share neither sequence nor structural similarity with the kinetoplastid-specific pATOM36 (36 kD). However, recent studies have shown that expression of pATOM36 can restore the growth and OM protein biogenesis defects in a yeast strain deleted for both Mim1 and Mim2 [20]. Moreover, in the converse experiment, simultaneous expression of Mim2/Mim2 in a *T*. *brucei* cell line ablated for pATOM36 was able to restore the OM protein biogenesis defect observed in these cells [20]. However, expression of the yeast proteins could not complement the growth arrest caused by the lack of pATOM36. This can be explained by the fact that Mim1/Mim2 only complement the OM protein biogenesis defect but not the impairment of mitochondrial DNA segregation caused by a deficient TAC that lacks pATOM36, both of which are essential processes.

Therefore, the MIM complex and pATOM36 are functional analogues that arose by convergent evolution. The successful reciprocal complementation experiments and the fact that Mim1/Mim2, as well as pATOM36, each are found in a protein complex that has the same molecular weight irrespectively of whether its subunits are expressed in the original or the heterogenous system demonstrate that the MIM complex and pATOM36 function autonomously. Furthermore, the restricted phylogenetic occurrence of the MIM complex—in fungi but not in their sister group the metazoans—and the fact that pATOM36 is specific for trypanosomatids suggest that the need to have a dedicated factor that facilitates insertion and/or assembly of N-terminally anchored mitochondrial OM proteins arose relatively late in evolution. Moreover, the observation that the import receptors Tom20 and Tom70 but not the MIM complex are conserved between fungi and metazoans suggests that the latter evolved later than the receptors.

## One or two TIM complexes?

**Fig 3:**
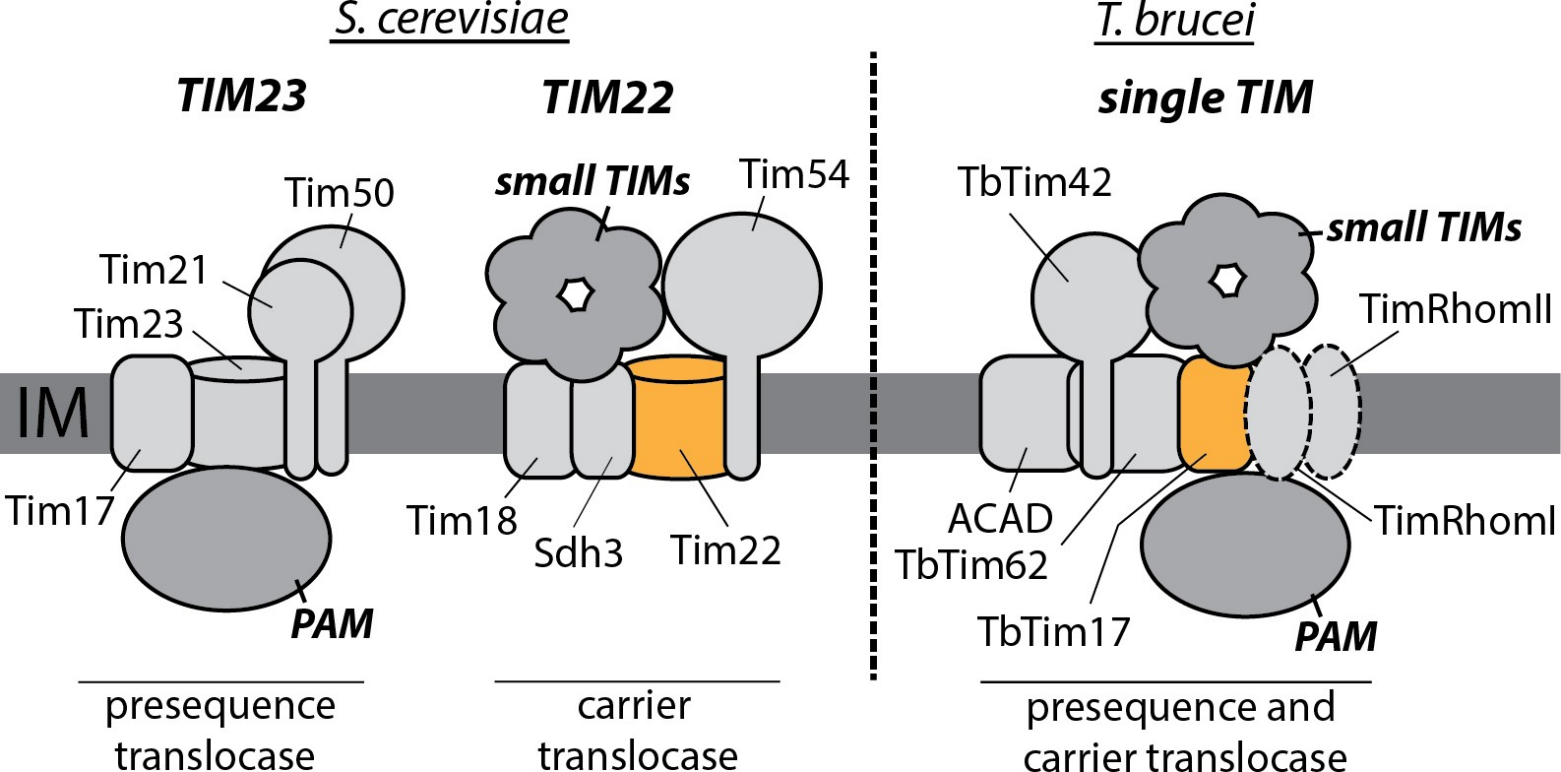
Comparison of the inner membrane protein translocases TIM23 and TIM22 of *S*. *cerevisiae* with the single *T*. *brucei* TIM complex. Tim22 and TbTim17 are orthologues and are indicated in orange. Other subunits are shown in light grey. The peripherally associated small TIM and the PAM complexes are indicated in dark grey. The composition of the trypanosomal PAM is unknown at present. The two inactive rhomboid-like proteases, TimRhomI and TimRhomII, were specific for the presequence translocase. The depiction of the trypanosomal TIM complex is schematic; its exact architecture remains to be elucidated. ACAD, acyl-CoA dehydrogenase; IM, inner membrane; PAM, presequence-associated motor; Sdh, succinate dehydrogenase; TIM, translocase of the IM.

The yeast mitochondrial inner membrane has two heterooligomeric protein complexes with nonoverlapping subunit composition, termed translocase of the inner membrane 23 (TIM23) and TIM22 [2, 4, 5]. They mediate import of either presequence-containing precursors (TIM23) or insertion of inner membrane proteins having multiple transmembrane regions, such as mitochondrial carrier proteins (TIM22) (Fig 3).

Using immunoprecipitations of tagged substrate proteins that were locked in the import channel, it was shown that trypanosomes—in contrast to yeast—have a single TIM complex only, that with minor compositional variations mediates import of both presequence-containing as well as carrier proteins [21]. Compositional analysis of the single trypanosomal TIM complex reveals that it consists of six integral membrane proteins of which only TbTim17 shows homology to any subunits of the yeast TIM23 and TIM22 complexes, respectively [22] (Fig 3). TbTim17, like Tim23 and Tim17 of the yeast TIM23 complex and Tim22 of the yeast TIM22 complex, belong to the Tim17/22/23 protein family. In yeast, Tim23, probably together with Tim17, forms the pore for presequence-containing proteins whereas Tim22 acts as the channel for carrier proteins. Bioinformatic analysis shows that TbTim17, despite its name, is most closely related to the Tim22 subfamily of proteins. The single trypanosomal TIM complex must have at least one protein-conducting pore. Which TIM subunits form this pore is presently unknown, but TbTim17—based on its similarity to Tim22—is a prime candidate. Further essential integral membrane subunits of trypanosomal TIM complex are Tim62 [23], Tim42, acyl-CoA dehydrogenase (ACAD) and two inactive rhomboid-like proteins (TimRhom I and TimRhom 2), although the latter were not recovered in a pulldown of a tagged mitochondrial carrier proteins that was stuck in the import channel [21]. Except for Tim17, Tim22, and Tim23, none of the yeast TIM23 or TIM22 subunits shows any homology to trypanosomal Tim subunits.

However, in both yeast and trypanosomes, a metabolic enzyme was incorporated into the TIM complexes: succinate dehydrogenase subunit 3 in the yeast TIM22 complex [24] and ACAD into the single trypanosomal TIM complex [21], although it is presently not known whether the ACAD found in the TIM is required for protein import and whether it has retained enzymatic activity. Together with the mitochondrial matrix protease that processes presequences and that in plant consists of two subunits that are identical to the two core subunits of the cytochrome bc1 complex [25], this makes recruitment of metabolic enzymes to mitochondrial proteins import complexes a recurring theme.

## Conclusion

Considering that mitochondria are of monophyletic evolutionary origin, the mitochondrial protein import systems of yeast and trypanosomes show amazing differences. Comparative analysis of these unique properties allows to define the very basic features of protein import that are shared between all eukaryotes due to chemical and physical constraints and not due to common descent. Moreover, it provides insight into how the endosymbiontic ancestor of mitochondria was converted into an organelle. Finally, because mitochondrial protein import is a core essential process, the parasite-specific import factors might be exploited as drug targets.
